# Evaluating Ecological Quality Under Dredging Disturbance Using Multiple Macrobenthic Indices in Shellfish Farming Areas of Gamak Bay, South Korea

**DOI:** 10.3390/biology15090671

**Published:** 2026-04-24

**Authors:** Jian Liang, Shu-Ping Zhang, Xu Tian, Zeng-Feng Zhao, Jiang-Yi Sun, Xiao-Yan Zhang, Se-Hyun Choi, Long-Ying Pei, Chae-Woo Ma

**Affiliations:** 1 Experimental Teaching Demonstration Centre of Food Safety and Nutrition, Xinjiang Institute of Technology, Aksu 843100, China; 2 Aksu Institute of Apple, Xinjiang Institute of Technology, Aksu 843100, China; 3 Department of Information Engineering, Xinjiang Institute of Technology, Aksu 843100, China; 4 College of Ecology and Environment, Xinjiang University, Urumqi 830017, China; 5 School of Smart Water Conservancy Engineering, Xinjiang Institute of Technology, Aksu 843100, China; 6 School of Civil and Hydraulic Engineering, Ningxia University, Yinchuan 750021, China; 7 Fisheries Business Team, Korea Fisheries Infrastructure Public Agency, Seoul 08588, Republic of Korea; 8 Department of Biology, Soonchunhyang University, Asan 31538, Republic of Korea

**Keywords:** macrobenthic index, South Sea of Korea, macrobenthos, shellfish, ecological quality status

## Abstract

Shellfish aquaculture can alter sediment conditions and affect benthic ecosystem functioning, so dredging is widely applied as a management strategy to mitigate sediment deterioration. However, its ecological effectiveness remains uncertain. This study evaluated ecological quality under the disturbance of dredging in shellfish farming areas of Gamak Bay, South Korea, using multiple macrobenthic indices. Macrobenthic samples and environmental data were collected before (May 2025) and after dredging (August 2025). Five macrobenthic indices, including the AZTI Marine Biotic Index (AMBI), BENTIX, Benthic Polychaete/Amphipod ratio (BPA), Benthic Pollution Index (BPI), and Multivariate AMBI (M-AMBI), along with a composite index, were used to assess ecological quality. Temporal changes within groups were tested using Wilcoxon signed-rank tests, and differences between dredged and control stations were examined using Mann–Whitney U tests. Multivariate analyses were used to explore environmental gradients and community responses. Results showed clear seasonal variation in environmental conditions and macrobenthic community structure. Most indices indicated a decline in ecological quality after dredging, with higher AMBI values and lower BENTIX, BPI, and M-AMBI values at dredged stations. However, these changes were not statistically significant (*p* > 0.05), suggesting limited short-term effects of dredging. The proportion of stations with acceptable ecological status decreased slightly from May to August. Seasonal factors, particularly temperature and salinity, played a dominant role in structuring benthic communities. Overall, the findings indicate that the short-term dredging effects were weaker than seasonal environmental variability. A multi-index approach is recommended for robust ecological assessment, and long-term monitoring is necessary to fully evaluate the effectiveness of dredging in shellfish aquaculture systems.

## Simple Summary

Shellfish farming is an important source of food, but it can lead to the accumulation of waste in seabed sediments, potentially harming marine life. Dredging, a process that removes surface sediments, is often used to improve environmental conditions in aquaculture areas. In this study, we investigated how dredging affected the ecological conditions of the seabed in a shellfish-farming area in South Korea. We collected samples before and after dredging and assessed changes in macrobenthos. The results showed that environmental conditions and biological communities changed over time, but these changes were primarily driven by seasonal factors, such as temperature, rather than by the dredging itself. Although some signs of environmental decline were observed after dredging, the overall effects were small and not statistically strong. This suggests that dredging alone may not quickly improve environmental quality. Our findings highlight the importance of long-term monitoring and more comprehensive management strategies to ensure sustainable shellfish farming and protect marine ecosystems.

## 1. Introduction

Shellfish aquaculture plays a critical role in global food security by providing a sustainable source of high-quality protein and essential nutrients [1,2]. As demand for seafood continues to rise, shellfish farming has expanded rapidly, particularly in coastal regions where environmental conditions are favorable for bivalve production [3,4]. Beyond its contribution to food supply, shellfish aquaculture also supports coastal economies and livelihoods. However, intensive farming practices can lead to the accumulation of organic matter, biodeposits, and associated contaminants in sediments, including nutrients such as nitrogen and phosphorus, sulfides, trace metals, and organic pollutants, thereby altering benthic habitats and compromising ecosystem health [5,6]. Therefore, balancing production efficiency with environmental sustainability has become a key challenge in modern shellfish aquaculture systems [7].

Macrobenthic communities are widely recognized as sensitive indicators of environmental change in marine ecosystems [8,9]. Due to their limited mobility, relatively long life cycles, and direct exposure to sediment conditions, macrobenthos respond in an integrated manner to multiple stressors, including organic enrichment, hypoxia, and contamination [10,11]. Changes in their community structure, diversity, and functional composition can reflect both short-term disturbances and long-term ecological degradation. Consequently, macrobenthic indices, such as the AZTI Marine Biotic index (AMBI) and its multivariate extension (M-AMBI), have been extensively used to assess ecological quality status (EcoQs) within environmental monitoring programs [12,13]. Compared to physicochemical measurements alone, these biological indices provide a more comprehensive evaluation of ecosystem functioning and disturbance gradients.

In South Korea, shellfish aquaculture is a major component of coastal fisheries, with numerous farming areas distributed across semi-enclosed bays characterized by high productivity and complex hydrodynamics [14,15]. Regions such as Gamak Bay have been intensively used for shellfish cultivation, leading to increased organic loading and sediment deterioration in some areas [16]. Previous studies have reported spatial heterogeneity in benthic responses across Korean coastal systems, typically occurring over distances of hundreds of meters to several kilometers, and often reflecting contrasts between farming and non-farming areas as well as differences among sub-regions of the bay, such as more enclosed inner zones and relatively well-flushed central basins [17,18]. Variations in hydrodynamic conditions, sediment characteristics, and anthropogenic pressures primarily drive these spatial patterns. Despite the ecological importance of these systems, systematic assessments that integrate multiple macrobenthic indices in shellfish farming areas remain relatively limited, particularly for management interventions aimed at environmental restoration [19,20].

Dredging is commonly employed as a management strategy to mitigate sediment degradation and improve benthic habitat conditions in aquaculture areas [21,22]. By removing organically enriched surface sediments, dredging is expected to reduce sulfide accumulation, enhance oxygen penetration, and facilitate the recovery of benthic communities [23]. However, its ecological effectiveness remains controversial, as dredging may also disturb the sediment structure and resuspend contaminants, leading to variable outcomes depending on local environmental conditions. In this context, a robust evaluation framework is required to quantify the ecological effects of dredging and to inform sustainable management practices. Therefore, the present study aims to assess ecological quality under the disturbance of dredging in shellfish farming areas of Gamak Bay, South Korea, using multiple macrobenthic indices. By integrating biological indicators with environmental gradients, this study seeks to provide a comprehensive understanding of benthic ecosystem responses and contribute to evidence-based management of aquaculture environments.

## 2. Materials and Methods

### 2.1. Study Area

Gamak Bay is located along the southern coast of South Korea, between 34°33′–34°45′ N and 127°38′–127°45′ E. The bay has an average depth of approximately 9 m, with a maximum depth of about 40 m in the southern basin. During summer, the water column in the basin is strongly stratified [16]. The presence of a thermocline restricts vertical mixing between surface and bottom waters, which may lead to hypoxic conditions in the bottom layer. Dissolved oxygen concentrations in bottom waters generally remain below 10 mg L^−1^ throughout the year, and hypoxic conditions (approximately 3 mg L^−1^) are particularly evident in the northwestern basin during summer [24]. Seasonal variation in water temperature ranges from 5.7 °C in winter to 24.2 °C in summer, while salinity ranges from 30.0 PSU to 33.6 PSU [24]. Sediments in the study area are predominantly fine-grained, with mean grain sizes ranging from 7.07 to 9.22 μm. The northern and central regions are mainly composed of silty clay and clayey silt, respectively. In contrast, small amounts of gravelly mud are distributed near the tidal channels at the bay mouth. Aquaculture activities in Gamak Bay are extensive, with oyster farming as the dominant cultivation practice throughout the bay [24].

### 2.2. Experimental Design

**Figure 1:**
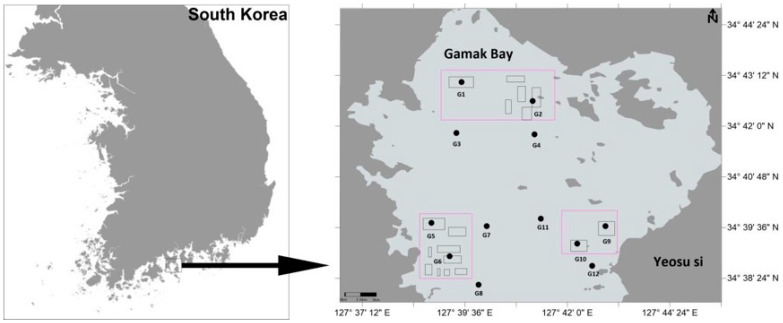
Study area and sampling sites in Gamak Bay, South Korea, showing shellfish farming areas and dredging zones. Note: Black rectangles indicate shellfish farming areas, and red-outlined regions represent dredging zones.

To improve environmental conditions in shellfish farming areas, dredging operations were conducted in Gamak Bay in June 2025 as a short-term intervention. The dredging activities were carried out over several consecutive days within designated shellfish farming zones and were not repeated during the study period. A combination of bottom trawling and grab dredging was employed to remove approximately the upper 20 cm of surface sediments, aiming to eliminate accumulated organic matter, shell debris, and aquaculture-related waste. For comparative purposes, six undisturbed control stations (G3, G4, G7, G8, G11, and G12) were established within the bay. These control sites were selected to have water depths and sediment characteristics similar to those of the dredged areas. They were located at least 300 m from the dredging sites to minimize direct mechanical disturbance (Figure 1).

Previous studies have shown that sediment plumes generated by bottom trawling and dredging can be transported over distances ranging from several hundred meters to kilometers, depending on the hydrodynamic conditions. For example, near-bottom dredging plumes may extend approximately 700–1200 m down-current, while resuspended sediments from trawling can be advected over even greater distances under certain conditions. In addition, modeling studies of shellfish dredging have reported plume influence ranges of approximately 260–540 m in moderate hydrodynamic settings [25]. Therefore, while the 300 m buffer distance adopted in this study falls at the lower end of reported plume dispersal distances and is expected to substantially reduce direct disturbance, it may not eliminate indirect hydrodynamic effects such as sediment resuspension and transport. However, turbidity and sediment resuspension were not continuously monitored during the dredging operations, and thus these effects were not quantitatively assessed.

### 2.3. Sample Collection

Sampling was conducted during spring tides in May and August 2025. At each station, macrobenthic samples were collected using a 0.05 m^2^ Van Veen grab sampler. Four replicate grabs were taken per station to ensure adequate representation of the benthic community. The collected sediments were sieved through a 0.5-mm mesh, and the retained organisms were preserved in 10% buffered formalin for subsequent laboratory identification and enumeration. Additional sediment samples for physicochemical analyses were obtained using the same grab sampler. Surface sediments (0–5 cm) were carefully subsampled and stored in clean polyethene containers. Samples for organic matter and nutrient analyses were kept at 4 °C in the dark and transported to the laboratory within 24 h. Sediment samples intended for geochemical analyses (acid volatile sulfide and total organic carbon) were stored at −20 °C until analysis to minimize biochemical alteration. Bottom water samples were collected using a Niskin water sampler deployed approximately 0.5–1.0 m above the sediment–water interface at each station. Water sampling was conducted at the same location and time as sediment grab collection to ensure comparability between the physicochemical and benthic data. At each station, in situ measurements of dissolved oxygen (DO), temperature, salinity, and pH were obtained using a multiparameter water quality sonde (YSI 6600, Yellow Springs Instruments, Yellow Springs, OH, USA). The probe was lowered to the near-bottom layer corresponding to the water sampling depth. Measurements were taken in triplicate and averaged to minimize instrumental variability. To account for potential water column stratification, vertical profiles of temperature and dissolved oxygen were recorded at each station at 1 m intervals from the surface to the bottom before sample collection. Bottom water conditions were defined based on these profiles. Stratification effects, particularly oxygen depletion in near-bottom waters, were considered when interpreting benthic responses, as vertical gradients in temperature and oxygen influence biogeochemical processes and ecological patterns in aquatic systems. Water samples collected using the Niskin sampler were transferred into pre-cleaned bottles and stored in insulated containers before further analysis.

### 2.4. Sample Analysis

Macrobenthic organisms were sorted and identified under a stereomicroscope (Leica M65 C, Leica Microsystems GmbH, Wetzlar, Germany). Taxonomic identification was performed independently by two experienced taxonomists to ensure accuracy and consistency, and specimens were identified to the lowest possible taxonomic level, typically to species level. After identification, all samples were preserved in 80% ethanol for long-term storage.

Sediment physicochemical parameters were analyzed using standardized procedures. Acid volatile sulfide was determined using the diffusion method. Sediment chemical oxygen demand was measured using the potassium permanganate oxidation method. Total organic carbon was analyzed using a total organic carbon analyzer (TOC-L, Shimadzu Corporation, Kyoto, Japan) after the removal of inorganic carbon with hydrochloric acid. Ignition loss (IL, %) was determined by combusting dried sediment samples in a muffle furnace (SX2-4-10, Yiheng Scientific Instrument Co., Ltd., Shanghai China) at 550 °C for 4 h. Mean grain size (φ) was measured using a laser particle size analyzer (Mastersizer 2000, Malvern Panalytical Ltd., Malvern, UK).

For bottom water samples, chemical oxygen demand was determined using the alkaline potassium permanganate method with an automated continuous-flow analyzer (QuAAtro, SEAL Analytical GmbH, Norderstedt, Germany).

### 2.5. Index Calculation

#### 2.5.1. Macrobenthic Index

To evaluate ecological quality status (EcoQs) in the study area, five widely used macrobenthic indices were applied: the AZTI Marine Biotic index (AMBI), BENTIX, BPA, the Benthic Pollution index (BPI), and Multivariate AMBI (M-AMBI). The computational approaches and corresponding classification thresholds for each index were based on previously established methodologies [26,27,28,29,30].

AMBI was calculated from the relative abundances of species assigned to five ecological groups (EGI–EGV), ranging from disturbance-sensitive to opportunistic taxa. The index integrates the weighted contribution of each ecological group to reflect environmental disturbance gradients. BENTIX was derived by grouping species into three categories (GI–GIII), representing sensitive, tolerant, and opportunistic assemblages, and calculating a weighted ratio between these groups.

BPA was computed from the relative frequencies of polychaetes and amphipods, reflecting shifts in community structure in response to organic enrichment. BPI was calculated based on functional feeding groups, including filter feeders, deposit feeders, carnivores, and opportunistic species, with predefined weighting coefficients to quantify pollution levels. M-AMBI integrates AMBI, species richness (S), and Shannon diversity (H′) into a multivariate framework, providing a more comprehensive assessment of ecological quality. For all indices, ecological quality status was classified into five categories (High, Good, Moderate, Poor, and Bad) according to established threshold values. Detailed computational formulas and classification criteria are provided in Supplementary Table S1.

#### 2.5.2. Composite Index

The composite index was derived from the EcoQS classifications generated by the five macrobenthic indices. For a simpler interpretation, the original five ecological quality categories were consolidated into two broader classes: acceptable (high and good status) and unacceptable (moderate, poor, and bad status) [29]. Each benthic index classified as acceptable contributed one point to the composite score. Stations with a total score greater than or equal to four (≥4) were classified as having an acceptable ecological quality status.

### 2.6. Statistical Analysis

Sampling stations were classified into dredged stations (G1, G2, G5, G6, G9, and G10) and control stations (G3, G4, G7, G8, G11, and G12) based on their dredging status in August.

For each macrobenthic index, temporal variation between May and August was examined within each group using paired comparisons, in which values from the same sampling stations were matched across the two sampling periods. For stations with replicate samples, replicate measurements were averaged to obtain a single representative value per station and time point before statistical analysis. Differences in individual macrobenthic indices between groups and sampling periods were assessed using Mann–Whitney U tests and Wilcoxon signed-rank tests, respectively. In addition, to evaluate whether dredging influenced the magnitude of temporal changes, differences (Δ = August − May) were calculated for each benthic index at each station, and these Δ values were compared between dredged and control stations using Mann–Whitney U tests.

Spearman’s rank correlation analysis was performed to evaluate relationships between benthic indices and environmental variables, including water-column parameters and sediment characteristics. To account for multiple comparisons, *p*-values from correlation analyses were adjusted using the Benjamini–Hochberg false discovery rate (FDR) procedure, and adjusted *p*-values (q-values) < 0.05 were considered statistically significant. Principal component analysis (PCA) was conducted to explore multivariate patterns in environmental variables and identify the main environmental gradients influencing benthic ecological conditions. To statistically evaluate overall differences in environmental conditions between groups and sampling periods, a permutational multivariate analysis of variance (PERMANOVA) was performed based on Euclidean distance matrices with 999 permutations.

To assess differences in macrobenthic community structure among groups, a canonical analysis of principal coordinates (CAP) was performed using Bray–Curtis similarity. Community data were standardized by total abundance and log(x + 1) transformed before analysis. CAP was conducted using predefined groups (dredged vs. control stations) in May and August, and leave-one-out cross-validation was applied to evaluate classification performance.

To further examine the relationships between macrobenthic community structure and environmental variables, a distance-based linear model (DistLM) was applied based on Bray–Curtis similarity. Before analysis, species abundance data were standardized by total abundance within each sample to account for differences in sampling effort, and subsequently log(x + 1) transformed to reduce the influence of highly abundant taxa. Environmental variables were selected using a stepwise selection procedure, and only variables with significant contributions (*p* < 0.05) were retained. These selected variables were then used to perform a distance-based redundancy analysis (dbRDA) to visualize relationships between community composition and environmental drivers. All statistical analyses were performed using GraphPad Prism (version 10.4.2) and R software (version 4.0).

## 3. Results

### 3.1. Environmental Characteristics in Subtidal Zones of Gamak Bay

The environmental conditions in the subtidal zone of Gamak Bay are summarized in Table 1. Seawater temperature ranged from 17.88 to 28.09 °C, with a mean value of 23.16 ± 4.07 °C, showing a clear seasonal pattern. Temperatures were similar between shellfish farming and control stations in May (19.28 ± 0.43 vs. 19.12 ± 0.67 °C), but increased markedly in August (27.14 ± 0.33 vs. 27.28 ± 0.54 °C).

Salinity ranged from 27.83 to 32.79 PSU (mean 30.40 ± 2.19 PSU), with higher values observed in May (~32.5 PSU) and lower values in August (~28.2 PSU), indicating strong seasonal variation but minimal differences between station types. Similarly, pH remained relatively stable across all stations and seasons (8.10–8.30).

Dissolved oxygen (DO) concentrations ranged from 6.20 to 7.37 mg/L (mean 6.88 ± 0.29 mg/L). DO values were slightly higher in May (approximately 7.00 mg/L) than in August (approximately 6.70–6.76 mg/L), with no clear differences between shellfish farming and control stations. In contrast, organic matter and sediment-related variables exhibited pronounced spatial and temporal variability. Water-column COD ranged from 0.26 to 2.92 mg/L (mean 1.44 ± 0.70 mg/L), with higher values observed in shellfish farming areas in May (2.00 ± 0.78 mg/L) compared to control stations (1.05 ± 0.60 mg/L). Sediment AVS ranged from 0.01 to 0.43 mg/g (mean 0.10 ± 0.10 mg/g), with notably higher values in shellfish farming stations in May (0.18 ± 0.14 mg/g), indicating localized reducing conditions associated with organic enrichment. In August, AVS values decreased across all stations. Sediment COD showed substantial variability, ranging from 3.92 to 37.75 mg/g, with higher values in May (approximately 16.1 mg/g) than in August (approximately 8.0 mg/g), suggesting seasonal accumulation and subsequent reduction in organic matter.

Total organic carbon (TOC) ranged from 4.25 to 17.37 mg/g (mean 8.10 ± 3.64 mg/g), with higher concentrations generally observed in shellfish farming stations, particularly in May (10.29 ± 4.20 mg/g), compared to control stations (6.75 ± 2.72 mg/g). Ignition loss (IL) ranged from 5.01% to 11.05% (mean 7.06 ± 1.74%), showing a pattern similar to TOC, with higher values in shellfish-farming areas and in May. Mean grain size ranged from 2.26 to 5.32 φ (mean 4.24 ± 0.97 φ), with relatively finer sediments observed at control stations in May (4.82 ± 0.58 φ) and coarser sediments in August.

**Figure 2:**
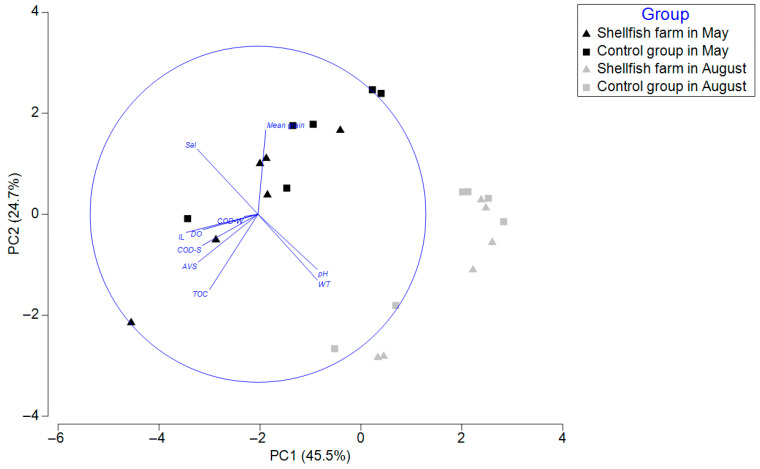
Principal component analysis (PCA) of environmental variables in the subtidal zone of Gamak Bay.

Principal component analysis (PCA) further illustrated the environmental heterogeneity among stations and sampling periods (Figure 2). The first two principal components explained 70.2% of the total variance, with PC1 and PC2 accounting for 45.5% and 24.7%, respectively. Samples collected in May were primarily distributed on the negative side of PC1. In contrast, those collected in August were generally positioned on the positive side, indicating a clear seasonal separation in environmental conditions.

Seawater temperature and pH were positively associated with PC1, suggesting a stronger contribution to the August samples. In contrast, salinity, ignition loss (IL), acid volatile sulfide (AVS), total organic carbon (TOC), and sediment chemical oxygen demand (COD-S) were oriented toward the negative side of PC1, indicating a greater association with the May samples and reflecting relatively higher levels of sediment organic enrichment during this period.

Consistent with the PCA results, PERMANOVA revealed a significant effect of sampling time on environmental variables (Pseudo-F = 8.43, *p* = 0.001). In contrast, no significant effect of dredging was detected (Pseudo-F = 0.82, *p* = 0.514) (Table 2).

### 3.2. Composition of Macrobenthic Communities

**Figure 3:**
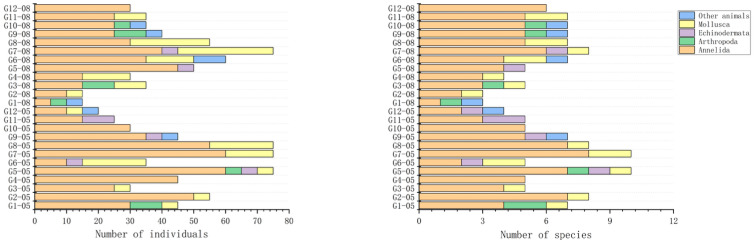
Composition of macrobenthic communities in terms of abundance and species richness across stations in May and August. Note: the suffixes “05” and “08” indicate samples collected in May and August, respectively. Stations G1, G2, G5, G6, G9, and G10 represent shellfish farming areas, while the remaining stations are control stations.

The composition of macrobenthic communities varied markedly among stations and between sampling periods (Figure 3). In both May and August, Annelida dominated the community in terms of both abundance and species richness across most stations, indicating their strong adaptability to local environmental conditions.

In May, several stations (e.g., G7–G9) exhibited relatively high total abundance and species richness, with substantial contributions from Mollusca and Arthropoda in addition to Annelida. The community included a mixture of ecological groups, with sensitive and indifferent species (EG I–II), such as *Paratapes undulatus* (EG I) and *Moerella hilaris* (EG I), co-occurring with tolerant and second-order opportunistic taxa (EG III–IV), including *Aricidea* spp. (EG III), *Hediste japonica* (EG III), and *Heteromastus filiformis* (EG IV) (Table S2). This pattern suggests relatively heterogeneous environmental conditions with moderate levels of organic enrichment.

In contrast, in August, a general decline in both total abundance and species richness was observed at several stations, particularly at G1 and G2, where the community structure became simplified and strongly dominated by Annelida. An increased contribution of opportunistic and tolerant species characterized this shift. Notably, taxa such as Capitella capitata (EG V), a well-known first-order opportunistic species, appeared in August, while second-order opportunistic and tolerant species (EG IV), including *Boccardia polybranchia*, *Heteromastus filiformis*, and Tharyx sp., became more prominent. In addition, several EG III taxa, such as *Aricidea* spp. and *Hediste japonica*, remained abundant across stations.

Meanwhile, stations without dredging influence maintained relatively more balanced community compositions, with contributions from multiple ecological groups (EG I–III) including species such as Paratapes undulatus and Magelona japonica (EG I). Overall, the observed shift toward higher proportions of EG IV–V taxa in August suggests a community response consistent with increased environmental stress and disturbance.

### 3.3. Group Discrimination of Macrobenthic Communities Based on CAP and PERMANOVA

**Figure 4:**
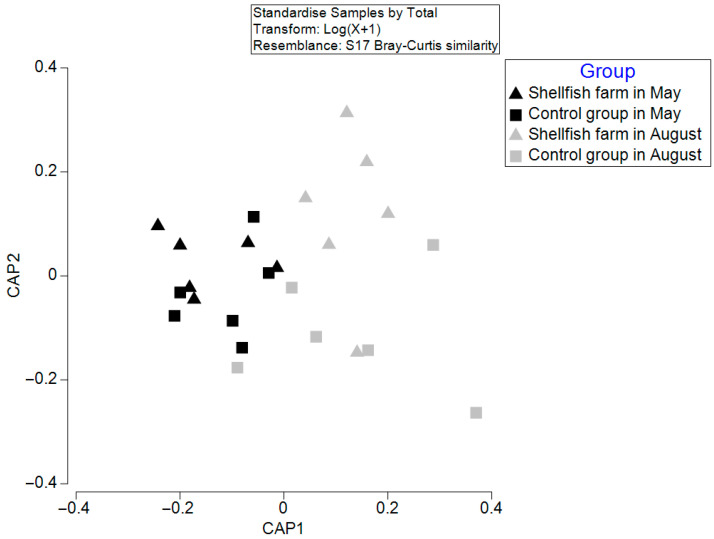
CAP ordination of macrobenthic community structure across groups based on Bray–Curtis similarity.

A Bray–Curtis similarity-based CAP revealed a moderate separation among the four groups (Figure 4). The first canonical axis (CAP1) explained the main pattern of variation, while the second axis (CAP2) provided additional but weaker separation.

In the ordination space, samples were primarily separated along CAP1 by sampling period, with most May samples on the negative side and most August samples on the positive side. This pattern indicates that temporal variation contributed substantially to differences in macrobenthic community structure. This observation was supported by PERMANOVA, which detected a significant effect of sampling time on community composition (Pseudo-F = 2.12, *p* = 0.021). In contrast, the effect of dredging was not statistically significant (Pseudo-F = 1.68, *p* = 0.085), although some degree of group separation was still visible in the ordination space (Table 3).

Within each sampling period, the separation between shellfish-farming and control stations was less distinct, particularly in May, when substantial overlap was observed. In August, a clearer but still partial differentiation among groups was evident, although some overlap remained. Overall, these results suggest that temporal variation was the primary factor structuring macrobenthic community patterns, while the influence of dredging was comparatively weaker and not statistically supported.

### 3.4. Environmental Drivers of Community Structure Based on dbRDA

**Figure 5:**
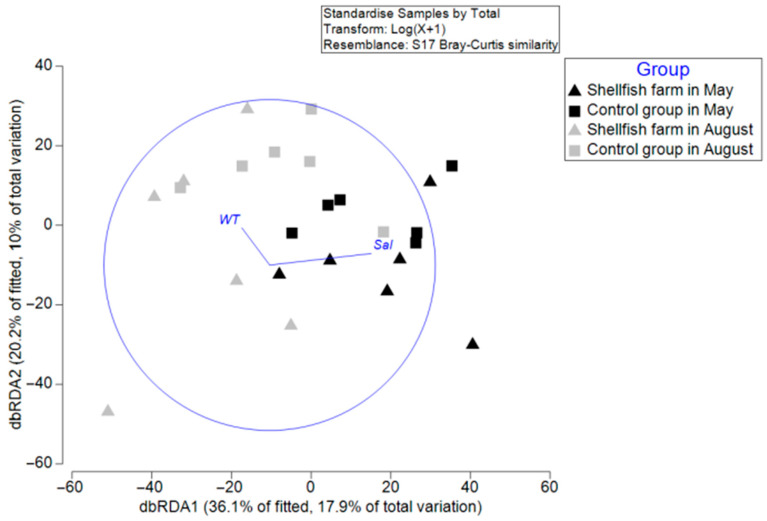
dbRDA ordination of macrobenthic communities, constrained by significant environmental variables (*p* < 0.05), based on Bray–Curtis similarity across shellfish farm and control sites in May and August. Note: WT, water temperature; Sal, salinity.

Distance-based redundancy analysis (dbRDA) revealed that a subset of environmental variables significantly explained the variation in macrobenthic community structure (Figure 5). The first dbRDA axis (dbRDA1) accounted for 36.1% of the fitted variation (17.9% of the total variation), while the second axis (dbRDA2) explained 20.2% of the fitted variation (10.0% of the total variation).

In the ordination space, samples were primarily separated along dbRDA1 by sampling period, with most May samples on the negative side and most August samples on the positive side, indicating a strong temporal structuring of community composition.

DistLM analysis identified seawater temperature (WT) and salinity (Sal) as significant predictors of community variation (*p* < 0.05; Table 4). Salinity was positively correlated with dbRDA1, indicating that higher salinity conditions were associated with shifts in community composition along the positive axis. In contrast, temperature showed a distinct directional influence, suggesting that it contributed independently to structuring the assemblages. Other variables, including pH and mean grain size, showed marginal contributions (*p* ≈ 0.05–0.10), while dissolved oxygen (DO), organic enrichment indicators (COD, TOC, IL), and AVS were not significant predictors (*p* > 0.05).

### 3.5. Results of Macrobenthic Indices

**Figure 6:**
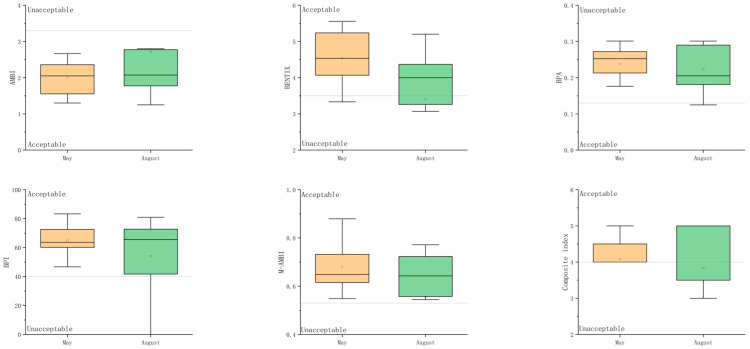
Seasonal variation in macrobenthic ecological indices between May and August. Note: The horizontal dashed lines indicate the threshold between acceptable and unacceptable ecological quality status (EcoQs). Boxes represent the interquartile range (IQR), central lines indicate medians, and whiskers represent the data range; orange boxes represent May, and green boxes represent August.

The boxplot analysis showed apparent but non-significant temporal differences in macrobenthic indices between May and August (Figure 6). AMBI values showed a slight increase in August compared with May, suggesting a tendency toward higher disturbance levels. In contrast, BENTIX showed a general decrease in August, suggesting a potential decline in ecological quality.

BPI displayed the most pronounced variability among all indices, with lower median values and a wider range in August, indicating substantial spatial heterogeneity following disturbance. Similarly, M-AMBI showed a slight decrease in August, although the overall variation remained relatively limited.

BPA exhibited relatively minor changes between the two sampling periods, suggesting that this index was less sensitive to temporal variation in the study area. The composite index also showed a slight decrease in August, consistent with the overall trend observed in BENTIX, BPI, and M-AMBI.

The proportion of stations classified as having acceptable ecological quality status (EcoQS) varied among macrobenthic indices and between sampling periods (Table 5). In May, AMBI and M-AMBI indicated that all stations (100.0%) were in acceptable condition, whereas BPI and BENTIX classified 91.7% and 83.3% of stations as acceptable, respectively. In contrast, BPA showed a substantially lower proportion, with only 9.1% of stations meeting the acceptable criterion.

In August, a general decline in the proportion of acceptable stations was observed across most indices. AMBI and M-AMBI decreased to 83.3%, while BENTIX declined to 66.7%. Similarly, BPI decreased to 75.0%. The composite index also decreased from 83.3% in May to 75.0% in August. BPA remained consistently low, with only 9.1% of stations classified as acceptable in both periods.

### 3.6. Results of Statistical Analysis

**Figure 7:**
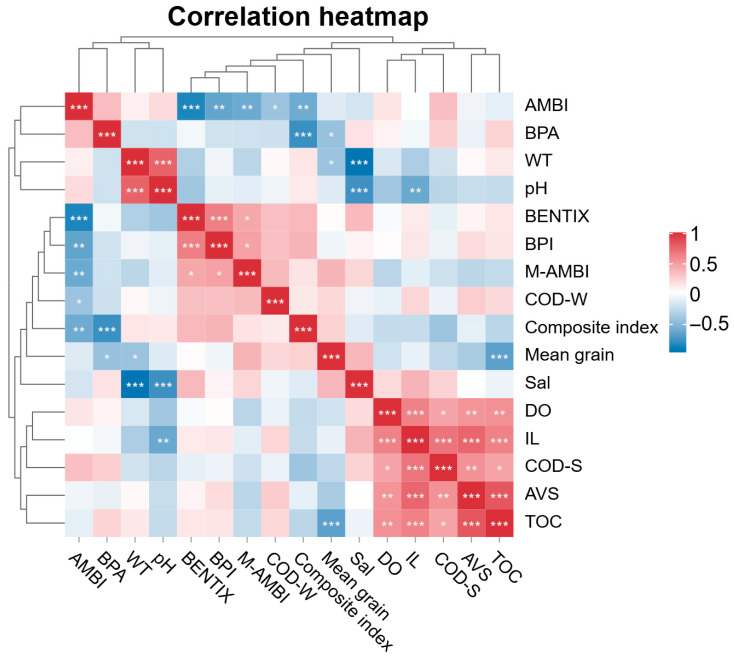
Correlation heatmap of macrobenthic indices and environmental variables in Gamak Bay. Note: Color intensity represents the strength and direction of Spearman’s rank correlation coefficients (red = positive, blue = negative). Asterisks indicate statistical significance (*p* < 0.05, *p* < 0.01, *p* < 0.001).

The correlation analysis revealed clear relationships between macrobenthic indices and environmental variables (Figure 7). Macrobenthic indices reflecting ecological quality, including BENTIX, BPI, M-AMBI, and the composite index, were positively correlated with one another, indicating consistent responses across indices. In contrast, AMBI showed negative correlations with these indices, reflecting its inverse relationship with ecological quality. Sediment-related variables, particularly TOC, AVS, IL, and sediment COD (COD-S), were strongly and positively correlated with one another, suggesting a common gradient of organic enrichment and reduced sediment conditions.

The macrobenthic indices showed different temporal responses between dredged and control stations (Table 6). In the dredged group, AMBI increased from 1.89 ± 0.50 in May to 3.08 ± 2.11 in August, whereas BENTIX, BPI, M-AMBI, and the composite index decreased from 4.73 ± 0.85 to 3.03 ± 2.03, 71.13 ± 8.15 to 45.82 ± 34.81, 0.71 ± 0.09 to 0.47 ± 0.35, and 4.00 ± 0.63 to 3.33 ± 1.97, respectively. In contrast, the control group showed relatively small temporal changes, with M-AMBI and the composite index remaining generally stable.

Within-group comparisons revealed no statistically significant temporal changes in any benthic index in either the dredged or control group (all *p* > 0.05) (Table 6). However, in the dredged stations, AMBI showed an increasing trend. In contrast, BENTIX, BPI, M-AMBI, and the composite index generally decreased from May to August, suggesting a tendency toward ecological deterioration following dredging.

To further evaluate whether dredging influenced the magnitude of temporal changes, the differences (Δ = August − May) in benthic indices were compared between dredged and control stations (Table 7). No statistically significant differences were detected between groups for any index (all *p* > 0.05). Among the indices, BPI exhibited the strongest response, decreasing by 25.32 ± 35.33 at dredged stations while increasing slightly by 3.38 ± 15.30 at control stations; this difference approached statistical significance (*p* = 0.093).

## 4. Discussion

### 4.1. Changes in Benthic Communities Before and After Dredging

The CAP analysis revealed a clear seasonal pattern in macrobenthic community structure, with communities more clustered in May and more dispersed in August (Figure 4), indicating relatively stable conditions in spring and increased heterogeneity in summer. The dbRDA results, supported by DistLM analysis, identified salinity (*p* = 0.003) and water temperature (*p* = 0.005) as significant environmental predictors of community structure (Figure 5; Table 4). Rather than indicating strict dominance, these results suggest that both variables played important roles in shaping macrobenthic assemblages. Seasonal differences in their relative influence likely reflect underlying hydrographic variability. In May, salinity was relatively high and stable at both the shellfish-farming (32.50 ± 0.12 PSU) and control stations (32.57 ± 0.12 PSU), which may have contributed to a consistent salinity gradient influencing species distribution under relatively stable environmental conditions. In contrast, during August, salinity decreased markedly (to approximately 28.20 ± 0.23 PSU in shellfish farming areas and 28.21 ± 0.21 PSU in control stations), while water temperature increased substantially (to approximately 27.14–27.28 °C). These changes suggest that seasonal hydrographic shifts, particularly warming and freshening, may have contributed to increased variability in community structure.

In addition, other environmental variables associated with sediment quality and organic enrichment, including DO, TOC, AVS, IL, and COD, did not indicate increased stress conditions in August. On the contrary, several of these variables were lower than in May, suggesting that organic enrichment and sediment-related stress were not intensified during the summer. Therefore, the observed community variability is more likely linked to physical environmental changes rather than a general deterioration of sediment quality. Previous studies in Korean coastal waters have shown that semi-enclosed bays are particularly vulnerable to summer hypoxia due to thermal stratification and terrestrial inputs [31,32]. Under such conditions, bottom-water dissolved oxygen can decline, influencing macrobenthic communities. However, in the present study, no clear evidence of intensified hypoxia was detected during August based on the measured variables. Nevertheless, seasonal warming may still contribute to increased metabolic demand, enhanced decomposition of organic matter, and localized oxygen depletion at the sediment–water interface, which can influence benthic organisms [33,34]. Such processes may increase community variability and promote patchy distributions without necessarily producing strong signals in bulk environmental measurements [35]. In addition, previous research in Gamak Bay has indicated that seasonal environmental variability can partially override the effects of management interventions, such as dredging, leading to community patterns that are more strongly associated with natural hydrographic conditions [36].

Overall, the results indicate that seasonal hydrographic variability, particularly increases in temperature and reductions in salinity, is a key driver of macrobenthic community dynamics in Gamak Bay. However, it should be noted that the present study was based on two sampling periods (May and August 2025), with dredging conducted in June 2025. Therefore, the May data represent pre-dredging conditions rather than an undisturbed baseline, as long-term shellfish aquaculture may have already influenced the sediment characteristics and community structure. Thus, the baseline condition in this study should be interpreted as an already modified state shaped by ongoing aquaculture activities, rather than a pristine reference condition. Given this limitation, the observed changes cannot be attributed solely to dredging and are more likely to reflect the combined effects of seasonal environmental variability and short-term post-dredging responses. Consequently, the ecological effects of dredging should be interpreted within the broader context of both seasonal fluctuations and existing anthropogenic influences.

### 4.2. Changes in Ecological Quality Before and After Dredging

In this study, five macrobenthic indices, together with one composite index, which have been widely validated in Korean coastal ecosystems, were applied to assess ecological quality [14,37,38]. Although these indices are based on different theoretical frameworks and calculation approaches, their assessment results showed a high level of consistency. Except for the BPA index, the proportion of stations classified as having “acceptable” ecological quality status exceeded 75% in Gamak Bay during both May and August. The BPA index is primarily calculated from the relative abundances of amphipods and polychaetes and is designed to reflect shifts in community structure along disturbance gradients [39,40]. Although this index has proven effective in specific contexts, such as oil spill events and organically enriched environments, its applicability to Korean coastal ecosystems appears limited. In the present study, BPA showed weaker agreement with the other indices, suggesting that it may not adequately capture the complexity of benthic responses in shellfish-farming areas. Therefore, it is not recommended to use the BPA index as a standalone indicator for ecological quality assessment in Korean coastal waters. The Benthic Health index (BHI), derived from the Infaunal Trophic index (ITI), has been used to evaluate ecological quality status in shellfish aquaculture areas in South Korea [41]. However, like BPA, the BHI is largely based on polychaete assemblages and does not incorporate responses from other important taxonomic groups. Considering the ecological complexity of shellfish farming environments, where multiple stressors such as organic enrichment, sulfide accumulation, and hydrodynamic variability interact, an index based on a single taxonomic group is unlikely to provide a comprehensive and accurate assessment of ecological conditions [42,43]. Therefore, reliance on a single index may lead to biased or incomplete conclusions. A multi-index approach that integrates indices with varying ecological sensitivities and theoretical foundations is better suited to capturing the multidimensional responses of benthic ecosystems [44,45,46]. This approach enhances the robustness of ecological assessments and reduces uncertainty associated with individual indices, thereby providing a more reliable basis for environmental management and decision-making.

No significant differences were detected among macrobenthic indices in both the analysis of changes before and after dredging and the comparison between dredged and control stations (Table 6 and Table 7). The observed patterns in dredged sites may reflect a limited short-term ecological response following dredging. Previous studies have shown that short-term changes following dredging may represent apparent rather than effective recovery of benthic communities [47]. Given that sampling was conducted approximately two months after dredging (June to August), the time interval may have been insufficient for substantial community changes or recovery processes to develop fully. Therefore, the ecological effects of dredging in Gamak Bay may be relatively weak or not yet fully expressed at the time of sampling. One possible explanation is that Gamak Bay is a semi-enclosed coastal system that has been subject to long-term shellfish aquaculture. Continuous organic enrichment and sediment disturbance associated with aquaculture may have already altered the baseline conditions of the benthic ecosystem, resulting in a relatively homogenized community structure [48,49]. Under such conditions, the relative effect of dredging may be less pronounced, making it difficult to detect significant differences using conventional benthic indices. Another important factor is the timing of post-dredging sampling [50]. In this study, the survey was conducted approximately two months after dredging, which may be insufficient to capture meaningful ecological recovery or community restructuring. Previous studies have shown that benthic recovery following dredging is a time-dependent process, often involving initial colonization by opportunistic species, followed by gradual succession toward a more stable community structure. Reported recovery times vary widely across studies, typically ranging from several months to multiple years, depending on sediment type, hydrodynamic conditions, and dredging intensity. For example, noticeable recolonization may occur within 6–12 months, whereas full recovery of community structure may take several years in some systems [51,52]. However, these timelines are not directly comparable to the present study, as recovery rates are strongly context-dependent. In Gamak Bay, a semi-enclosed aquaculture system with relatively fine sediments and pronounced seasonal variability, the recovery trajectory is likely to be influenced by both local environmental conditions and ongoing anthropogenic pressures. Therefore, the absence of significant differences observed in this study is more likely to reflect an early transitional stage of ecological response rather than a true lack of dredging effects.

These findings highlight the importance of incorporating longer-term monitoring and temporal replication in future studies to better capture the dynamics of benthic recovery. In addition, combining macrobenthic indices with other approaches, such as functional trait analysis or sediment biogeochemical measurements, may improve the sensitivity of ecological assessments in complex aquaculture environments.

### 4.3. Limitations of the Study and Management Recommendations

Despite the valuable insights provided by this study, several limitations should be acknowledged. First, the present analysis was based on two sampling periods, conducted in May and August, representing late spring and summer conditions, respectively. Although these periods capture important seasonal stages characterized by high biological activity and organic matter accumulation, they may not fully reflect the annual variability in macrobenthic communities and sediment conditions. Seasonal dynamics, particularly during winter or transitional periods, may influence community recovery processes and ecological quality assessments following dredging. Second, the temporal scale of post-dredging assessment remains relatively short. Macrobenthic communities often require extended periods to re-establish stable structures after physical disturbance, and short-term responses may not necessarily indicate long-term ecological recovery. Therefore, caution should be exercised when interpreting the effectiveness of dredging based solely on short-term observations. Third, although multiple macrobenthic indices were applied to improve the robustness of the assessment, each index has inherent limitations and may respond differently to specific environmental stressors. The integration of additional approaches, such as functional trait analysis or multivariate ecological modeling, could further enhance the mechanistic understanding of benthic responses. Future management strategies should emphasize long-term monitoring, adaptive management, and the integration of ecological indicators with environmental variables. Such approaches will be essential to ensuring the sustainable development of shellfish aquaculture and maintaining benthic ecosystem integrity in coastal environments.

## 5. Conclusions

This study provides a comprehensive evaluation of ecological quality in the shellfish farming areas of Gamak Bay under the effects of dredging disturbance, using multiple macrobenthic indices and integrated statistical approaches. The results consistently showed that although most benthic indices indicated a tendency toward ecological deterioration in dredged areas after intervention, these changes were not statistically significant. This suggests that the short-term ecological effects of dredging were relatively limited. In contrast, strong seasonal variability, particularly associated with temperature increase and salinity shifts, exerted a more pronounced influence on macrobenthic community structure and ecological quality. These findings highlight that natural environmental fluctuations, especially summer conditions, may override the immediate effects of management interventions such as dredging. Among the indices applied, the high consistency among most indices, together with the composite index, supports the reliability of a multi-index framework for ecological quality assessment in aquaculture systems. From a management perspective, the results suggest that dredging alone may not be sufficient to achieve rapid ecological improvement in semi-enclosed bays. Instead, its effectiveness should be evaluated within a broader temporal context that accounts for seasonal dynamics and site-specific environmental conditions. Therefore, long-term monitoring, combined with multi-index assessment approaches, is essential for developing sustainable management strategies in shellfish aquaculture areas.

## Figures and Tables

**Table 1:** Environmental data in the subtidal zones of Gamak Bay.

Environment Data	Total (Mean ± SD)	May (Mean ± SD) (Shellfish Farming)	May (Mean ± SD) (Control Stations)	August (Mean ± SD) (Shellfish Farming)	August (Mean ± SD) (Control Stations)
Seawater temperature, °C	17.88–28.09 (23.16 ± 4.07)	16.62–19.89 (19.28 ± 0.43)	17.88–19.76 (19.12 ± 0.67)	26.80–27.59 (27.14 ± 0.33)	26.34–28.09 (27.28 ± 0.54)
Salinity, PSU	27.83–32.79 (30.40 ± 2.19)	32.37–32.68 (32.50 ± 0.12)	32.47–32.79 (32.57 ± 0.12)	27.83–28.42 (28.20 ± 0.23)	27.87–28.42 (28.21 ± 0.21)
pH	8.10–8.30 (8.16 ± 0.06)	8.10 (8.10 ± 0)	8.10 (8.10 ± 0)	8.16–8.30 (8.21 ± 0.05)	8.16–8.30 (8.22 ± 0.05)
DO, mg/L	6.20–7.37 (6.88 ± 0.29)	6.85–7.16 (7.00 ± 0.11)	6.75–7.37 (7.02 ± 0.23)	6.20–7.12 (6.70 ± 0.33)	6.20–7.32 (6.76 ± 0.40)
COD, mg/L	0.26–2.92 (1.44 ± 0.70)	0.99–2.92 (2.00 ± 0.78)	0.26–1.93 (1.05 ± 0.60)	0.85–2.17 (1.61 ± 0.49)	0.53–2.17 (1.56 ± 0.60)
AVS, mg/g	0.01–0.43 (0.10 ± 0.10)	0.03–0.43 (0.18 ± 0.14)	0.01–0.23 (0.07 ± 0.08)	0.02–0.09 (0.06 ± 0.03)	0.02–0.24 (0.09 ± 0.08)
COD, mg/g	3.92–37.75 (12.51 ± 8.5)	4.42–37.75 (16.17 ± 12.31)	4.78–29.71 (16.09 ± 10.24)	3.92–12.48 (8.26 ± 3.48)	3.92–12.77 (7.88 ± 3.41)
TOC, mg/g	4.25–17.37 (8.10 ± 3.64)	7.21–17.37 (10.29 ± 4.20)	4.51–11.66 (6.75 ± 2.72)	6.03–15.58 (8.82 ± 3.78)	6.03–12.28 (7.80 ± 2.31)
IL, %	5.01–11.05 (7.06 ± 1.74)	6.16–11.05 (9.00 ± 1.67)	5.53–9.22 (7.14 ± 1.32)	5.01–6.80 (5.71 ± 0.84)	5.01–8.35 (6.01 ± 1.37)
Mean grain, ∮	2.26–5.32 (4.24 ± 0.97)	2.90–5.30 (4.15 ± 0.87)	3.70–5.30 (4.82 ± 0.58)	2.26–4.80 (3.65 ± 1.19)	2.75–4.80 (4.00 ± 0.88)

Note: AVS, acid volatile sulfide; COD, chemical oxygen demand; DO, dissolved oxygen; TOC, total organic carbon.

**Table 2:** PERMANOVA results for environmental variables testing the effects of dredging and sampling time.

Source	Degrees of Freedom	Sum of Squares	Mean Square	Pseudo-F	*p* (perm)
Dredging	1	5.4	5.4	0.82	0.5
Sampling time	1	55.4	55.4	8.43	0.001

**Table 3:** PERMANOVA results for macrobenthic communities testing the effects of dredging and sampling time.

Source	Degrees of Freedom	Sum of Squares	Mean Square	Pseudo-F	P (perm)
Dredging	1	5055.3	5055.3	1.68	0.085
Sampling time	1	6402.8	6402.8	2.12	0.021

**Table 4:** DistLM results revealing the influence of environmental data on macrobenthic community structure.

Environment Data	Sum of Squares	Pseudo-F	*p*
Seawater temperature, °C	8640.2	2.8	0.005
Salinity, PSU	9112	2.9	0.003
pH	6079	1.9	0.051
DO, mg/L	3901.8	1.2	0.338
COD, mg/L	2263.5	0.6	0.759
AVS, mg/g	1976.4	0.6	0.828
COD, mg/g	3152.9	0.9	0.503
TOC, mg/g	4509.9	1.4	0.194
IL, %	5387.8	1.6	0.092
Mean grain, ∮	6077.3	1.8	0.055

Note: AVS, acid volatile sulfide; COD, chemical oxygen demand; DO, dissolved oxygen; TOC, total organic carbon.

**Table 5:** Proportion of stations classified as acceptable ecological quality status (EcoQS) based on different macrobenthic indices in May and August.

Index	May	August
AMBI	100%	83.3%
BENTIX	83.3%	66.7%
BPA	9.1%	9.1%
BPI	91.7%	75.0%
M-AMBI	100.0%	83.3%
Composite index	83.3%	75.0%

**Table 6:** Temporal changes in macrobenthic indices within the dredged and control stations (May vs. August).

Group	Index	May (Mean ± SD)	August (Mean ± SD)	Change	*p* Value
Dredged	AMBI	1.89 ± 0.50	3.08 ± 2.11	1.18 ± 2.31	0.625
	BENTIX	4.73 ± 0.85	3.03 ± 2.03	−1.70 ± 2.54	0.219
	BPA	0.24 ± 0.07	0.24 ± 0.06	0.01 ± 0.08	0.688
	BPI	71.13 ± 8.15	45.82 ± 34.81	−25.32 ± 35.32	0.156
	M-AMBI	0.71 ± 0.09	0.47 ± 0.35	−0.23 ± 0.41	0.438
	Composite index	4.00 ± 0.63	3.33 ± 1.97	−0.67 ± 2.66	0.875
Control	AMBI	2.12 ± 0.47	2.35 ± 0.40	0.23 ± 0.74	0.688
	BENTIX	4.33 ± 0.73	3.77 ± 0.50	−0.57 ± 1.03	0.219
	BPA	0.24 ± 0.05	0.20 ± 0.06	−0.04 ± 0.10	0.344
	BPI	58.77 ± 8.84	62.15 ± 18.08	3.38 ± 15.30	0.562
	M-AMBI	0.66 ± 0.12	0.64 ± 0.06	−0.02 ± 0.11	1.000
	Composite index	4.17 ± 0.75	4.33 ± 0.82	0.17 ± 1.47	1.000

**Table 7:** Comparison of changes in benthic indices between the dredged and control stations.

Index	Dredged (Δ Mean ± SD)	Control (Δ Mean ± SD)	*p* Value
AMBI	1.18 ± 2.31	0.23 ± 0.74	0.937
BENTIX	−1.70 ± 2.54	−0.56 ± 1.03	0.818
BPA	0.01 ± 0.08	−0.04 ± 0.10	0.589
BPI	−25.32 ± 35.33	3.38 ± 15.30	0.093
M-AMBI	−0.23 ± 0.41	−0.02 ± 0.11	0.818
Composite index	−0.67 ± 2.66	0.17 ± 1.47	0.806

## Data Availability

The raw data supporting the conclusions of this article will be made available by the authors on request.

## Supplementary Materials

The following supporting information can be downloaded at: https://www.mdpi.com/article/10.3390/biology15090671/s1, Table S1: Computational approaches and reference thresholds employed for evaluating ecological quality status (EcoQs) using macrobenthos indices; Table S2: Macrobenthic species list in Gamak Bay.
