# Supplementary material for: Evaluating Ecological Quality Under Dredging Disturbance Using Multiple Macrobenthic Indices in Shellfish Farming Areas of Gamak Bay, South Korea
Source: Biology (Basel). 2026 Apr 24;15(9):671. doi: 10.3390/biology15090671 (PMC13162846; doi:10.3390/biology15090671)
Supplement: Supplementary file 1 [file biology-15-00671-s001.zip › Supplementary Material.pdf]

Supplementary Material

Evaluating Ecological Quality under Dredging Disturbance Using Multiple Macrobenthic Indices in Shellfish Farming Areas of Gamak Bay, South Korea

Jian Liang[1,2†], Shu-Ping Zhang[3,4†], Xu Tian[5], Zeng-Feng Zhao[6], Jiang-Yi Sun[1,2], Xiao-Yan Zhang[1,2], Se-Hyun Choi[7,8], Long-Ying Pei[1,2*], Chae-Woo Ma[8*]

1 Experimental Teaching Demonstration Centre of Food Safety and Nutrition, Xinjiang Institute of Technology, Aksu, China
2 Aksu Institute of Apple, Xinjiang Institute of Technology, Aksu, China
3. Department of Information Engineering, Xinjiang Institute of Technology, Aksu, China
4. College of Ecology and Environment, Xinjiang University, Urumqi, China
5 School of Smart Water Conservancy Engineering, Xinjiang Institute of Technology, Aksu, China
6 School of Civil and Hydraulic Engineering, Ningxia University, Yinchuan, Ningxia, China
7 Fisheries Business Team, Korea Fisheries Infrastructure Public Agency, Seoul, Republic of Korea
8 Department of Biology, Soonchunhyang University, Asan, Republic of Korea
* Correspondence: peilongy@163.com (P-L,Y) and cwooma@sch.ac.kr (C-W, M)
† These authors contributed equally to this work

Table S1. Computational approaches and reference thresholds employed for evaluating ecological quality status (EcoQs) using macrobenthos indices

Indices	Algorithm	Index values	EcoQs
AMBI	$= [(0 \times \%\ \text{EGI}) + (1.5 \times \%\ \text{EGII}) + (3 \times \%\ \text{EGIII}) +(4.5 \times \%\ \text{EGIV})(6 \times \%\ \text{EGV})]/100$	0.0-1.2<br>1.2-3.3<br>3.3-5.0<br>5.0-6.0<br>>6.0	High<br>Good<br>Moderate<br>Poor<br>Bad
BENTIX	$= [6 \times \%\ \text{G1} + 2(\%\text{G2} + \%\ \text{G3})]/100$	6-4.5<br>4.5-3.5<br>3.5-2.5<br>2.5-2.0<br>0.0	High<br>Good<br>Moderate<br>Poor<br>Bad
BPA	$= \log[(fP)/(fA + 1) + 1)]$	0-0.02452<br>0.02455-0.13002<br>0.13002-0.19884<br>0.19884-0.25512<br>>0.25512	High<br>Good<br>Moderate<br>Poor<br>Bad
BPI	$= [1 - (\text{a} \times \text{N1} + \text{b} \times \text{N2} + \text{c} \times \text{N3} + \text{d} \times \text{N4})/(\text{N1} + \text{N2} + \text{N3} + \text{N4})/\text{d}] \times 100$	60-100<br>40-60<br>30-40<br>20-30<br>0-20	High<br>Good<br>Moderate<br>Poor<br>Bad
M-AMBI	$= K + (\text{a} \times \text{AMBI}) + (\text{b} \times H') + (\text{c} \times \text{S})$	>0.77<br>0.53-0.77<br>0.38-0.53<br>0.20-0.38<br>≤0.2	High<br>Good<br>Moderate<br>Poor<br>Bad

Notes: For AMBI, EGI = disturbance-sensitive species; EGII = disturbance-indifferent species; EGIII = disturbance-tolerant species; EGIV = second-order opportunistic species; EGV = first-order opportunistic species. For BENTIX, GI = EGI + EGII; GII = EGIII + EGIV; GIII = EGV. For BPA, fP = the frequency of polychaetas; fA = the frequency of amphipods. For BPI, N1 = filter feeders or large carnivores; N2 = surface deposit feeders or small carnivores; N3 = subterranean deposit feeders; N4 = opportunistic species; a=0; b=1; c=2; d=3. For M-AMBI, S = number of species; H′= Shannon's diversity index; a, b, and c = coefficients.
